# Cadaver-based therapeutic endoscopic training for peroral endoscopic myotomy: a realistic model for skill acquisition

**DOI:** 10.1007/s12565-025-00914-6

**Published:** 2025-12-29

**Authors:** Maiko Tabuchi, Keiichi Hashiguchi, Hitomi Minami, Aya Wakamatsu, Kazunobu Saiki, Daisuke Endo, Yasuhiko Nakao, Hiroko Inomata, Junya Shiota, Taro Akashi, Moto Kitayama, Kayoko Matsushima, Naoyuki Yamaguchi, Keiko Ogami-Takamura, Hisamitsu Miyaaki, Yuko Akazawa

**Affiliations:** 1 Department of Histopathology and Cell Biology, Nagasaki University Graduate School of Biomedical Sciences, 1-12-4, Sakamoto, Nagasaki, 852-8523 Japan; 2 Department of Gastroenterology and Hepatology, Nagasaki University Graduate School of Biomedical Sciences, 1-7-1, Sakamoto, Nagasaki, 852-8501 Japan; 3 Department of Endoscopy, Nagasaki University Hospital, 1-7-1, Sakamoto, Nagasaki, 852-8523 Japan; 4 Tachibana Bay Clinic, 487-8 Aba, Nagasaki, 851-0123 Japan; 5 Department of Macroscopic Anatomy, Nagasaki University Graduate School of Biomedical Sciences, 1-12-4, Sakamoto, Nagasaki, 852-8523 Japan; 6 Center of Cadaver Surgical Training, School of Medicine, Nagasaki University, 1-12-4, Sakamoto, Nagasaki, 852-8523 Japan

**Keywords:** Achalasia, Cadaver, Peroral endoscopic myotomy, Endoscopy/education

## Abstract

The rarity of achalasia has led to limited opportunities for practical training in educational environments. Cadaver surgical training was developed to enhance surgical skills by providing physicians with experience comparable to that of on-the-job training using structures identical to living organs, thereby proving highly beneficial. Recently, cadavers have been preserved using the Thiel method, which maintains both mobility and organizational flexibility.　However, there are few reports on therapeutic endoscopy, including peroral endoscopic myotomy, in the context of cadaver surgical training. We investigated the feasibility of using cadaver surgical training for therapeutic endoscopy training, termed Cadaver-based Therapeutic Endoscopy Training, for peroral endoscopic myotomy. This study was conducted in 2024 at the Cadaver Surgical Center of Nagasaki University using four cadavers preserved using the Thiel method. We performed peroral endoscopic myotomies on three cadavers and evaluated the observability of the esophageal mucosa, penetration of the local injection fluid, feasibility of creating a submucosal tunnel, visibility of indocyanine green injection, and inner circular muscle incision. The layered structure of the digestive tract was well preserved in the Thiel-fixed cadavers, and peroral endoscopic myotomy was performed without major issues. Cadaver-based Therapeutic Endoscopy Training may serve as a realistic training model and may be beneficial for peroral endoscopic myotomy training.

**Supplementary Information:**

The online version contains supplementary material available at 10.1007/s12565-025-00914-6.

## Introduction

Peroral endoscopic myotomy (POEM) is an endoscopic procedure used to treat achalasia (Inoue et al. 2009). The rarity of achalasia poses significant challenges for medical trainees to gain adequate experience managing this condition (Gonzalez et al. 2023). According to the POEM training guidelines of the European Society of Gastrointestinal Endoscopy (Tate et al. 2025), at least 20 cases of ex vivo or animal training and at least 10 cases under expert supervision for initial human POEM, as on-the-job training (OJT), are needed (Tate et al. 2025). Human training involves learning through direct patient care. This step provides real-world experience and cultivates essential skills, such as managing bleeding or perforation, which are difficult to replicate through the simulator, although it can be stressful for trainees and involves potential risks. In addition, due to the low prevalence of achalasia, trainees often have limited opportunities to encounter and manage patients with achalasia during their training periods. Consequently, trainees may struggle to develop the skills and confidence necessary to effectively manage patients with achalasia.

To compensate for the scarcity and concern for safety in POEM training, ex vivo training and animal models have been the predominant approaches for initial training in endoscopic procedures, including POEMs (Gonzalez et al. 2023; Tate et al. 2025). However, the use of animal models poses several challenges, such as the need for ethical considerations regarding animal welfare and limited reproducibility due to anatomical differences.

Cadaver surgical training (CST) is a valuable training method used before OJT (Tominaga et al. 2022). This allows for repeated practice, error correction, and experimentation, fostering a deeper understanding of surgical procedures. The embalming method reported by Thiel utilizes a lower concentration of formalin (3–6%) than the conventional method (8–10%) and incorporates food-grade additives, such as propylene glycol and sodium bisulfite (Thiel 1992).　This technique preserves the texture and mobility of the skin, muscles, blood vessels, nerves, and ligaments. Therefore, the motility and flexibility of the organs are sufficiently maintained to perform surgery. Cadaver training on cadavers preserved via the Thiel method has been conducted since 2017 at Nagasaki University, with a focus on surgical training (Tominaga et al. 2022).

Although the utility of CST as an educational instrument is recognized within the surgical discipline, there are only a few studies addressing its application in endoscopic procedures (Balekuduru and Appaji 2021; Finocchiaro et al. 2021; Rohr et al. 2023). Furthermore, the human anatomy of cadavers ensures a consistent training experience and facilitates procedural skills applicable to actual clinical practice. To the best of our knowledge, there are no reports of advanced endoscopy methods, such as POEMs, in Thiel-fixed cadavers.

In this study, we evaluated whether POEM can be technically performed using cadaver-based therapeutic endoscopic training (CTET).

## Methods

In Japan, cadaver surgical training is legally permitted only at accredited medical universities and must be supervised by the Department of Anatomy in accordance with national regulations (Shichinohe et al. 2022). This study was conducted under these regulatory requirements.

In 2024, we conducted POEM on four cadavers preserved using the Thiel method. All cadavers underwent computed tomography (CT) scans before fixation to evaluate anatomical conditions. As this approach was newly implemented, the initial cadaver (Case 0, not included in the analysis) revealed a practical limitation: excessive esophageal residual contents related to the underlying disease impeded procedural progress.

This observation led to refinements in our selection process, and the subsequent cadavers (Cases 1–3) were selected with careful consideration of the cause of death and gastrointestinal residual contents to ensure optimal procedural conditions. The characteristics of all four cadavers are presented in the Supplemental Table.

The training was conducted using human cadavers donated for medical education, with full respect for donor dignity and the “Guidelines for Cadaver Dissection in Education and Research of Clinical Medicine” in Japan. The study protocol was reviewed and approved by the Ethics Committee of the Nagasaki University Graduate School of Biomedical Sciences (approval number: 21052801-2). Consent for CST was obtained from the donors and their families before death.

The equipment used in this study comprised a scope (GIF-H290T; Olympus, Tokyo, Japan) and a VIO 300D (AMCO, Tokyo, Japan) electrosurgical generator. The procedure used a Flush knife BT-S (Catalog number: DK2620JI-B15-, FUJIFILM, Tokyo, Japan), Triangle Tip Knife J (Catalog number: KD-645, Olympus, Tokyo, Japan), and normal saline stained with indigo carmine as the injection solution. The treatment devices used in this study were reused with a prior cleaning process.

The evaluation of the POEM procedure comprised observability of the mucosa, penetration of local injection fluid into the submucosal layer, feasibility of creating a submucosal tunnel, and visibility of indocyanine green (ICG) injection (Minami et al. 2015) (attempted in two cadavers: cases 2 and 3), and the ability to incise the circular muscles of the esophagus while leaving the longitudinal muscle intact. During POEM, it is customary to close the entry point into the submucosal tunnel using clips at the conclusion of the surgery. However, this step was not performed in this study due to the inability to secure consent from the bereaved families for the presence of foreign bodies.

### CTET for POEM

**Fig. 1:**
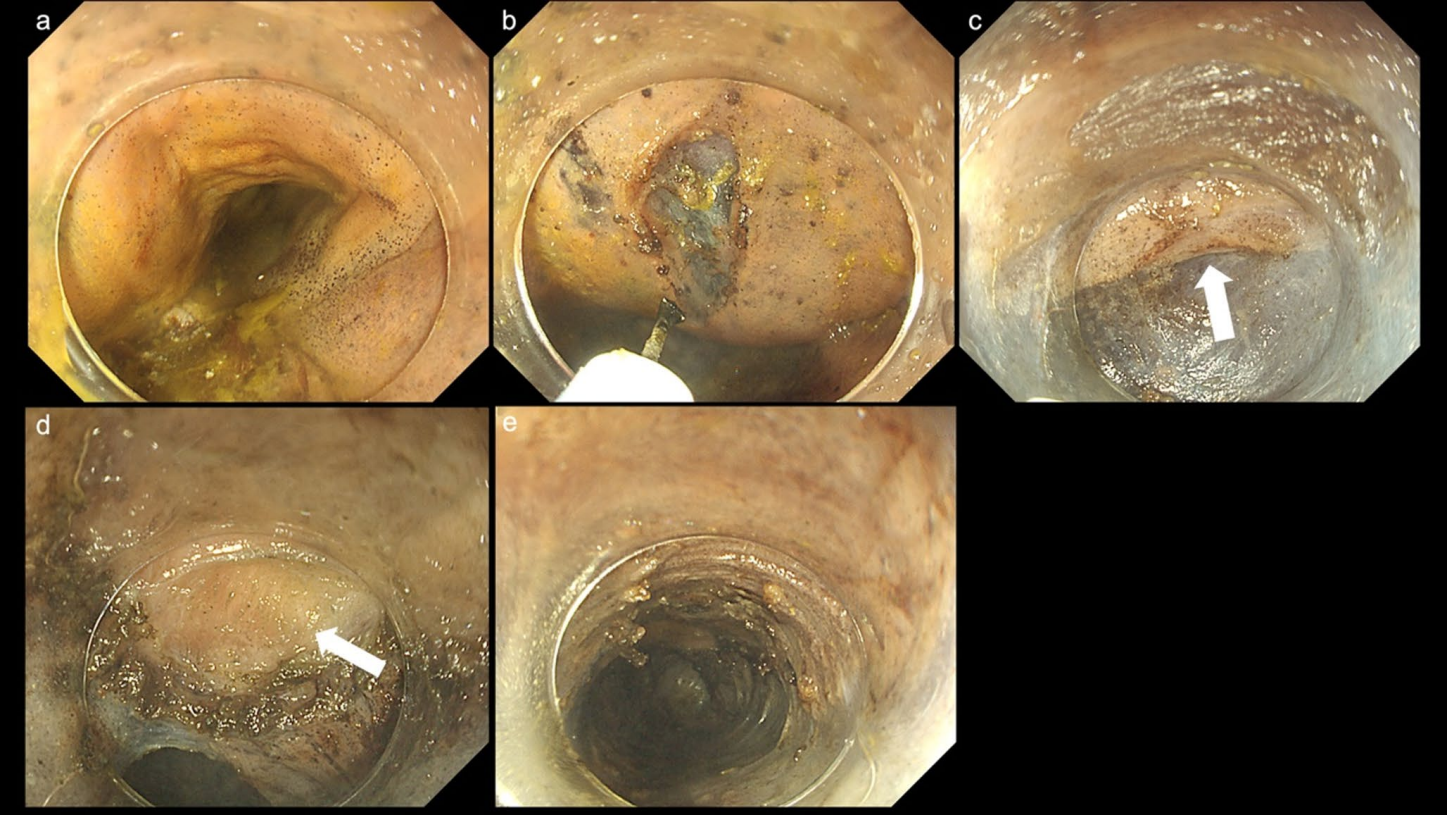
Endoscopic findings in cadaver case 1.** a** Esophageal mucosa.** b** Local injection of normal saline and subsequent mucosal incision.** c** The image illustrates the formation of a submucosal tunnel. The white arrow represents the inner circular muscle.** d** Endoscopic image of the circular and longitudinal muscles (white arrows).** e** The incision of the circular muscle was completed

**Fig. 2:**
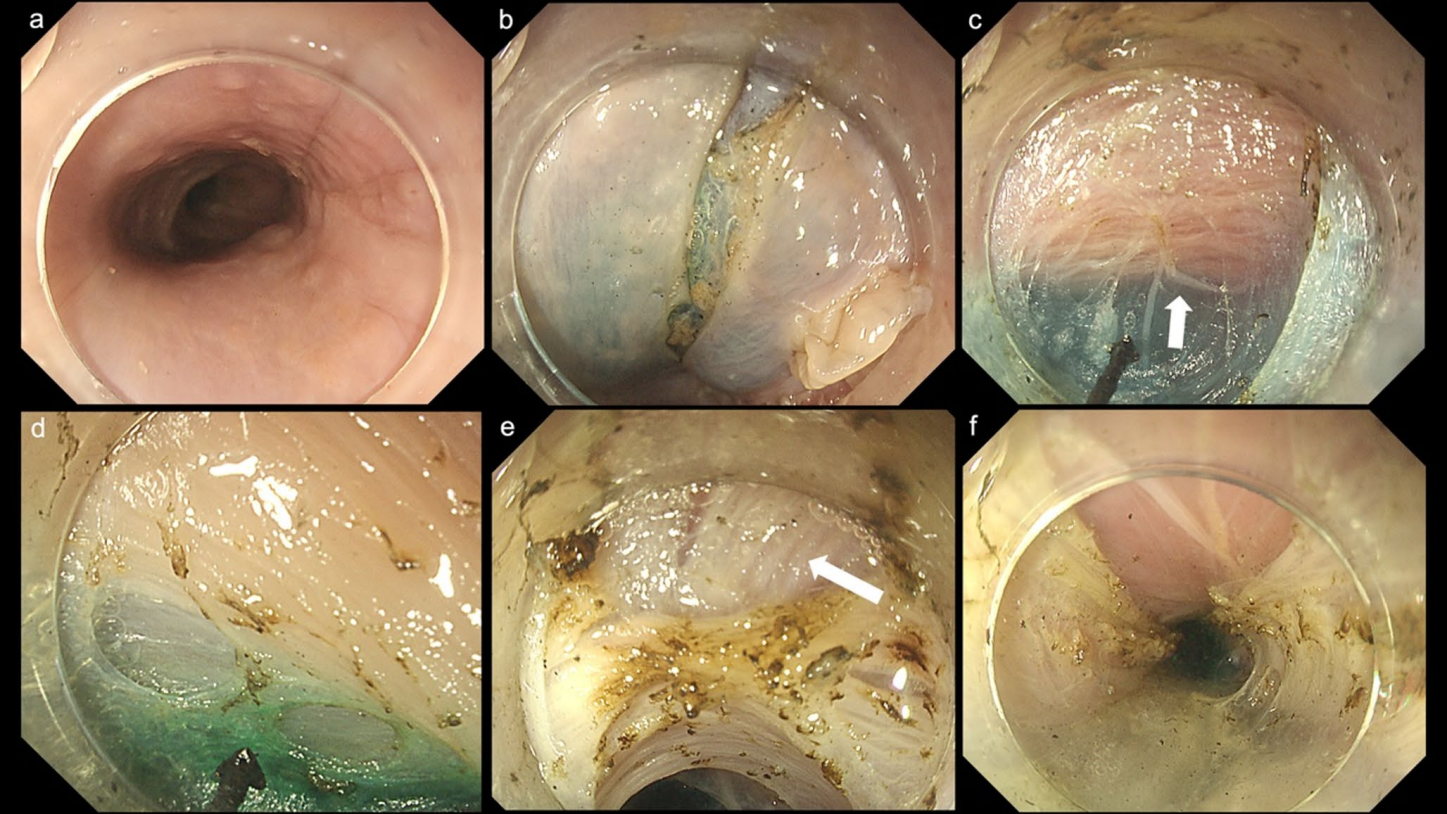
Endoscopic findings in cadaver case 2.** a** Esophageal mucosa.** b** Local injection of normal saline and subsequent mucosal incision.** c** The image illustrates the formation of a submucosal tunnel. The white arrow represents the inner circular muscle.** d** Image showing the green color of indocyanine green in the submucosal tunnel.** e** Endoscopic image revealing the incision of the circular muscle, thereby exposing the longitudinal muscle (white arrow).** f** The myotomy was successfully completed

**Fig. 3:**
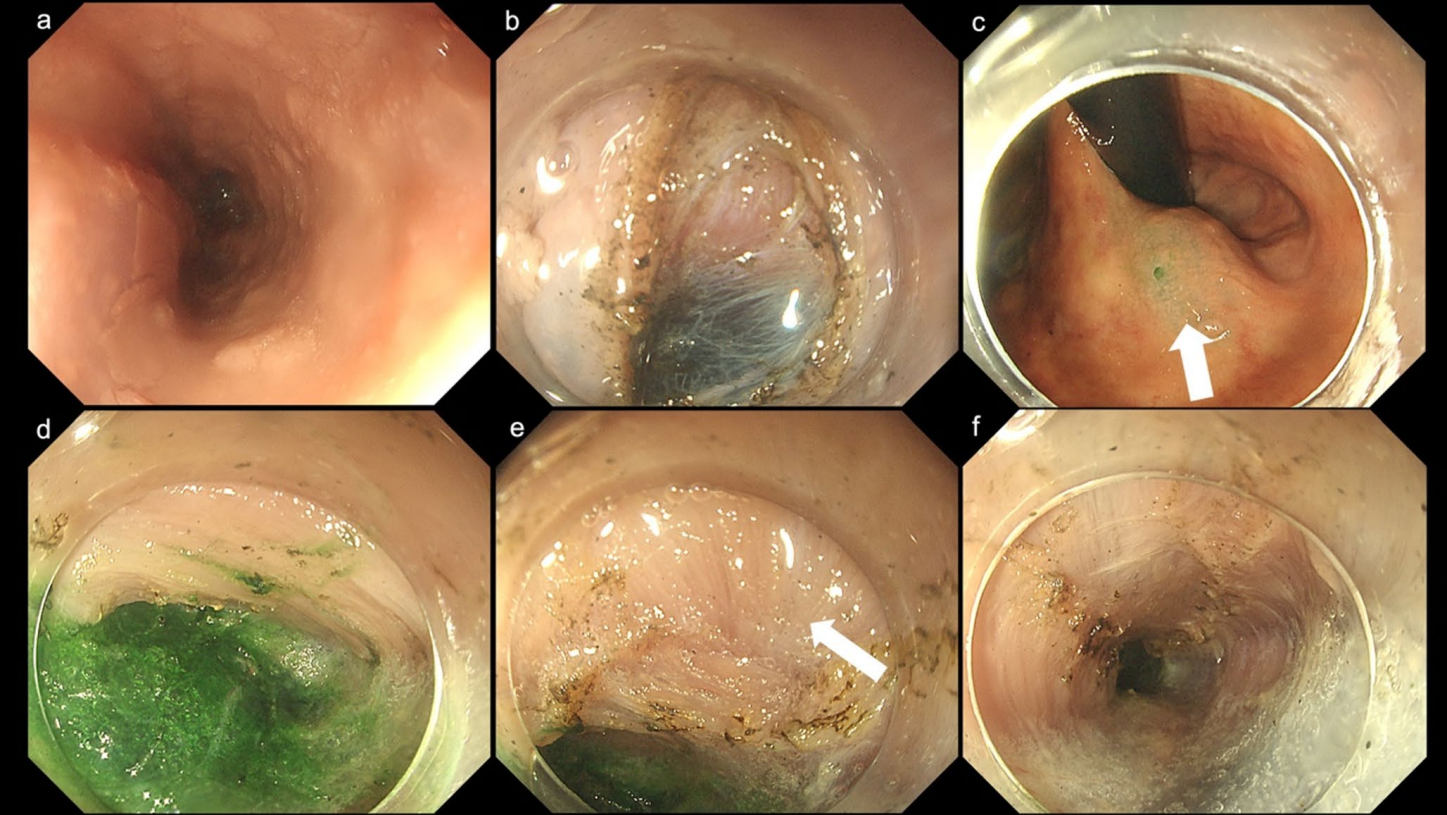
Endoscopic findings in cadaver case 3.** a** The mucosa of the esophagus was observed to be in a condition closely resembling that of a living organism, with no residual matter detected.** b** Following endoscopic injection of normal saline and subsequent mucosal incision, the submucosal layer becomes readily observable.** c** The image illustrates the indocyanine green injection at the gastric cardia to indicate the endpoint (white arrow).** d** Image showing the green color of the indocyanine green observed from the submucosal tunnel.** e** Endoscopic image revealing the incision of the circular muscle, thereby exposing the longitudinal muscle (white arrow).** f** The incision of the circular muscle was successfully completed

In three cases, the esophagus and stomach were confirmed to be empty (cases 1–3: each case shown in Figs. 1, 2 and 3, respectively), allowing POEM performance. However, in one case, POEM was not performed because the stomach was full due to intestinal obstruction.

In all three cases (cases 1–3), the esophageal mucosa was intact, permitting normal observation (Figs. 1a and 2a, and 3a). Although the pharyngeal section was stiff, we were able to insert the fiberscope into the esophagus. In Case 1, residual material was present during the initial observation; however, it was removed after applying water.

The submucosal injection was successfully performed in all three cadavers (Figs. 1b and 2b, and 3b). The integrity of the submucosal layer was preserved by using the Thiel method. Each layer, including the epithelium, submucosal layer, and muscular layer, was distinctly visualized and separated in a manner analogous to that observed in living tissues (Figs. 1c and 2c, and 3b). Subsequently, a submucosal tunnel was successfully established in all three cadavers. The green coloration of the ICG was clearly detectable as a benchmark by administering a local injection into the gastric cardia (Figs. 2d and 3c, and 3d) in the cadaveric cases (all ICG attempted cases, cases 2 and 3). The demarcation line between the submucosal layer and the inner circular muscle was clearly observable in all cases (Figs. 1c and 2c, and 3d). Subsequently, a myotomy of the circular muscle was performed. Separation of the circular and longitudinal muscle layers was successfully achieved in all three cases. We confirmed that the longitudinal muscle was well-preserved after the procedure (Figs. 1d and e, 2e and f and 3e, and 3f). A representative case (case 3) is shown in the POEM video (Video 1).

## Discussion

This pilot study confirms that POEM can be technically performed using CTET, suggesting possible utility in POEM training This is because our procedural cases confirmed that all mucosal layer structures were preserved using the Thiel method. In particular, the ability to incise only the circular muscle while preserving the longitudinal muscle, along with confirming gastric access using ICG, demonstrates the high utility of this model for POEM training. In this validation study, POEM was performed at the 2 o’clock position in all cases. However, it was possible to perform three POEM procedures per cadaver at the 2, 5, and 7 o’clock positions, allowing trainees to gain additional opportunities for experience.

The rarity of achalasia results in limited opportunities for trainees to practice POEMs. Thiel-fixed cadavers provide realistic anatomical representations, enabling trainees to familiarize themselves with human structures and variations. This is the first report to propose that CTET using POEM may significantly enhance the availability of ex vivo endoscopic treatment techniques.

Cadaver training has certain limitations, particularly in replicating hemostatic procedures and managing intraoperative complications. Moreover, the selection of suitable cadavers for endoscopic training requires careful evaluation of the cause of death and pre-assessment using CT imaging. Furthermore, after cadaver training, OJT offers an invaluable experience in terms of patient interaction, real-time decision-making, and adaptation to unforeseen circumstances, which are essential components of therapeutic endoscopy practices that cannot be entirely replicated in cadaver training with experts.

## Conclusions

CTET may serve as a realistic training model comparable to living organs and may be beneficial for POEM training.

## Supplementary Information

Below is the link to the electronic supplementary material.

Supplementary Material 1.

Supplementary Video.

## Data Availability

No datasets were generated or analysed during the current study.
