# Supplementary material for: Cadaver-based therapeutic endoscopic training for peroral endoscopic myotomy: a realistic model for skill acquisition
Source: Anat Sci Int. 2025 Dec 29;101(3):366–70. doi: 10.1007/s12565-025-00914-6 (PMC13179226; doi:10.1007/s12565-025-00914-6)
Supplement: Supplementary file 1 — Supplementary Material 1. [file 12565_2025_914_MOESM1_ESM.docx]

Supplemental Table. Characteristics or the cadavers

|  | Gender | Ages | Cause of death |
| --- | --- | --- | --- |
| Case 0* | Female | 90s | Digestive system disease |
| Case 1 | Female | 80s | Respiratory disease |
| Case 2 | Female | 70s | Metastatic malignant tumor |
| Case 3 | Female | 80s | Chronic neurological disease |

*Case 0 was excluded from this study.
